# Heterogeneity of the Dendrite Array Created in the Root of Cored SX Turbine Blades during Initial Stage of Crystallization

**DOI:** 10.3390/ma14010080

**Published:** 2020-12-26

**Authors:** Robert Paszkowski, Jacek Krawczyk, Włodzimierz Bogdanowicz, Dariusz Szeliga, Jan Sieniawski

**Affiliations:** 1 Institute of Materials Engineering, University of Silesia in Katowice, 1a 75 Pułku Piechoty St., 41-500 Chorzów, Poland; jacek.krawczyk@us.edu.pl (J.K.); wlodzimierz.bogdanowicz@us.edu.pl (W.B.); 2 Department of Materials Science, Rzeszow University of Technology, 2 W. Pola St., 35-959 Rzeszów, Poland; dszeliga@prz.edu.pl (D.S.); jansien@prz.edu.pl (J.S.)

**Keywords:** SX-superalloys, cored turbine blades, dendrite array, X-ray topography

## Abstract

The roots of cored single-crystalline turbine blades made of a nickel-based CMSX-4 superalloy were studied. The casts were solidified by the vertical Bridgman method in an industrial ALD furnace using the spiral selector and selector continuer situated asymmetrically in the blade root transverse section. Scanning electron microscopy, the Laue diffraction and X-ray diffraction topography were used to visualize the dendrite array and the local crystal misorientation of the roots. It has been stated that heterogeneity of the dendrite array and creation of low-angle boundaries (LABs) are mostly related to the lateral dendrite branching and rapid growth of the secondary and tertiary dendrites near the surface of the continuer–root connection. These processes have an unsteady character. Additionally, the influence of the mould walls on the dendrite array heterogeneity was studied. The processes of the lateral growth of the secondary dendrites and competitive longitudinal growth of the tertiary dendrites are discussed and a method of reducing the heterogeneity of the root dendrite array is proposed.

## 1. Introduction

Nickel-based superalloys, due to their unique strength properties, are used for the production of many parts of aircraft engines, in particular, high-pressure turbines blades. They must meet many requirements concerning their quality, for example heat and corrosion resistance and high mechanical strength for complex loads [1,2,3,4]. Currently in the aviation industry, the CMSX-4 superalloy is most often used for the production of the blades. The necessity of reducing the blades temperature during operation means that they are often produced with cooling bores [3,5]. The single-crystalline (SX) cored turbine blades are usually produced by the Bridgman method using the ceramic moulds. Directional crystallization by the Bridgman method allows one to obtain casts with a high homogeneity of parallel dendrite array and high homogeneity of the crystal orientation distribution. However, sometimes the homogeneity may be affected. The heterogeneity of the structure causes an increase in the local stress of the blades during operation which may contribute to their cracking.

**Figure 1:**
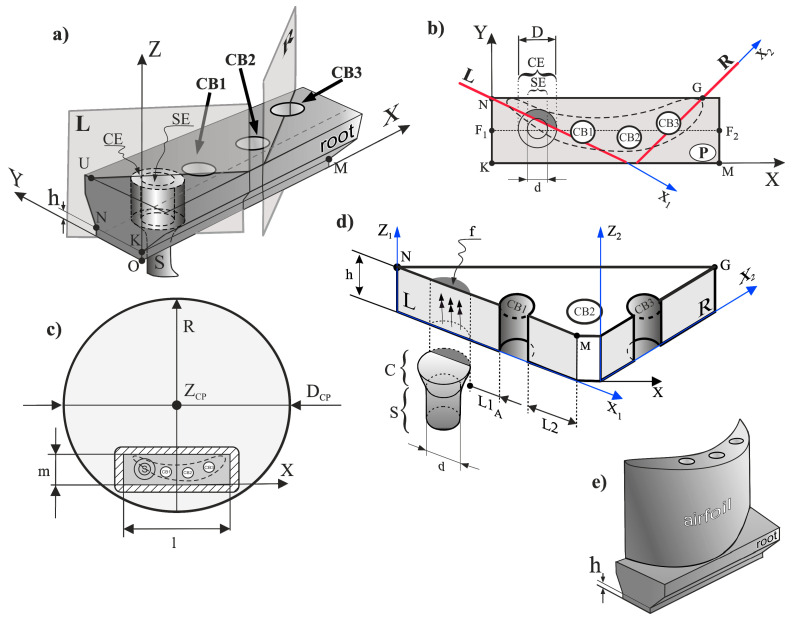
Scheme of the root and cutting planes L and R (**a**) and transverse section P of the root (plane NKM) (**b**), location of the casting mould on a chill-plate with D_CP_ diameter and a marked contour, as well as a scheme of the airfoil on the root (**c**) part of the root with thickness h obtained by cutting with planes L and R with marked location of the selector S and the continuer C (**d**) and illustration of the turbine blade (**e**). Z—root axis, CB1, CB2, CB3—cooling bores, SE and (CE)—selector extension and continuer extension in root, f—fragment of CE. The dashed line in (**b**,**c**) shows the location of the airfoil transverse section.

Attempts are often made to determine the reasons for heterogeneity of the dendrite array and the crystal orientation by production of the model casts with a simplified shape. As an example, the part with a narrow cross-section connected with the wide platform may be presented [6,7,8]. This part allows for the verification of the theoretically calculated temperature distribution or crystallization front shape, and consequently—creation of models of the dendrite array. A cast with a simplified shape in the form of a blade fragment is easier to analyze but the analysis does not take into account the possible interactions of the dendrites with the more complicated surfaces of the casting mould in the cored blades. In the present work we decided to produce SX casts of blade roots with cooling bores of a circular cross-section using a spiral selector with the continuer localized asymmetrically in the transverse section of the root (Figure 1a). Ceramic cores, passing axially parallel to the *Z* axis of root through the entire mould down to its bottom surface, were applied for formation of the cooling bores.

The most satisfactory strength properties of the SX turbine blades are observed when the axis Z (Figure 1a) is parallel to the [001] crystallographic direction. It has been stated that any deviation of this direction from the *Z* axis causes a deterioration in creep resistance [1]. The best creep resistance occurs in the [111] direction [1]. However, due to technical difficulties with obtaining the blades in this direction, the [001] direction is most often used [1]. The fact that the creep resistance is the best in the [111] direction may be related to the vibration mode of large amplitude in a defect-free anharmonic lattice [9]. The undisturbed growth of the dendrites with the cubic FCC structure takes place in the direction of [001], which is parallel to the withdrawal direction in the Bridgman method. However, the directional crystallization in a mould with a complex shape can lead to heterogeneity of the dendrite array and its crystal orientation [10,11,12]. This significantly affects the quality of the blade [1,13,14]. 

The SX casts obtained by the Bridgman method usually contain the selector and the continuer from which the secondary dendrites grow laterally in the root (parallel to the plane NKM (P), Figure 1a,b). The surfaces of the ceramic cores, that formed the cooling bores CB1, CB2, CB3, can disturb the lateral growth process that may cause heterogeneity of the dendrite array formed during crystallization in the root and heterogeneity of the dendrites’ crystal orientation. A change in the local crystal orientation, which would not be eliminated in subsequent production stages, for example in heat treatment [10], may be a consequence of the above mentioned heterogeneity. Even small local changes in the crystal orientation of the root created during crystallization may reduce the strength properties of the blades [15,16]. The heterogeneity of the dendrite array and crystal orientation created in the initial stage of the root crystallization, near the plane of the selector continuer–root connection, can be inherited by the whole root and airfoil [17]. For this reason, it is important to analyse the as-cast dendritic structure of the root parts that crystallize the earliest.

The aim of this studies was to analyse the heterogeneity of the dendrite array and its relation to local changes in the crystal orientation of the root areas that crystallize the earliest, i.e., located near the surface of the selector continuer–root connection of the single-crystalline cored turbine blades made of the CMSX-4 superalloy. 

## 2. Materials and Methods 

The SX cored blade roots made of commercial CMSX-4 nickel-based superalloy were obtained by directional solidification using the Bridgman technique. The roots were produced in the Research and Development Laboratory for Aerospace Materials, Rzeszów University of Technology, Poland, using the industrial (ALD Vacuum Technologies Inc., East Windsor, CT, USA) furnace [18]. A withdrawal rate of 3 mm/min was used.

The casts of cored roots consisted of the basic elements, such as the selector located asymmetrically in transverse-section of the root (Figure 1a) and the cooling bores, with the location imposed by the shape of the airfoil transverse section which could be crystallized with the root (Figure 1b,c). The selector was connected to the root by the continuer C (Figure 1b–d) with cross-sectional diameter D = 10 mm (Figure 1b,d) measured at the connection plane. The continuer extension (CE) is an area of the root limited by the projection of the continuer circumference parallel to the *Z* axis. The diameter of the selector channel cross-section (d in Figure 1d) was 6 mm. The part h of the root with thickness h = 5 mm (Figure 1a,d,e) was cut and studied. The bottom surface of part h, perpendicular to the *Z* axis was directly connected with the continuer. The dimensions *m* and *l* of the root transverse-section were 18 mm and 60 mm, respectively. The diameter D_CP_ of the chill-plate was equal 200 mm. The *Z* axis of the blade was parallel to the withdrawal direction. Three cooling bores with the circular cross-section and the diameter of 6 mm were parallel to the *Z* axis. The transverse microsection P (Figure 1b) was prepared from the upper surface of the part h.

**Figure 2:**
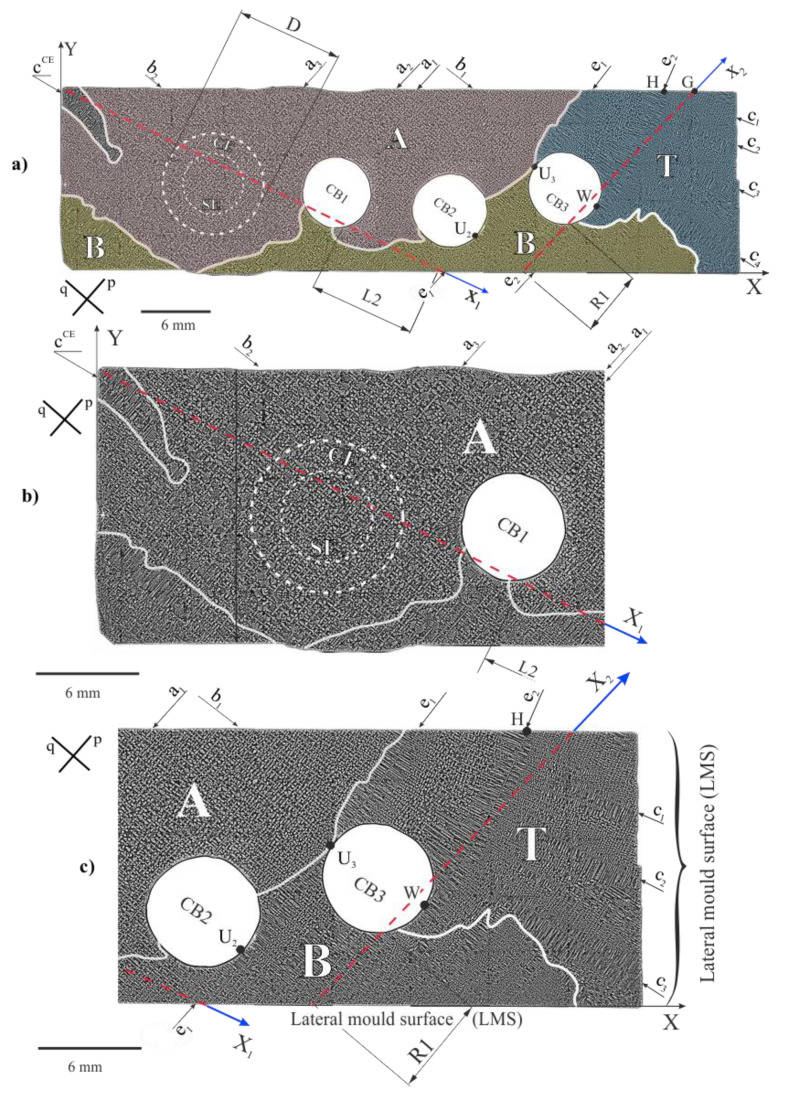
Dendritic structure visualized on the P section and the scheme of the structure division into the areas A, B and T (**a**); enlargement of the left (**b**) and right (**c**) fragments of (**a**); dashed lines connected with the axis X_1_ and X_2_ indicate traces of L and R—cutting planes; p and q—directions of the secondary dendrites.

After studying the microsection P, part h was cut along the L and R planes (Figure 1a,b) which were parallel to the *Z* axis. The planes of the section L and R were chosen in such a way that the L plane cuts the CE subarea and the cooling bore CB1, and the R plane cuts the cooling bore CB3, while additionally at the same time, the L and R planes (Figure 1d) were almost parallel to the secondary dendrite direction q and p (Figure 2). In this way, the sample with the shape shown in Figure 1d was obtained. All the microsections were prepared for analysis using the standard procedure for CMSX-4 superalloy [19]. 

The dendritic structure was visualised by scanning electron microscopy (SEM) with back-scattered electron imaging (BSE). The JSM-6480 JEOL microscope (JEOL Ltd., Tokyo, Japan) was used for investigations. The SEM macro-images of entire P, L and R (Figure 1b,d) sections were obtained by merging multiple separate SEM micro-images. The changes of the crystal orientation of the local areas and the low-angle boundaries (LABs) were visualized by the X-ray reflective topograms obtained from the P surface (Figure 1b). In order to obtain the topograms, it was necessary to define the crystal orientation of the h part. For this purpose, the Laue method was applied. The Laue patterns were obtained using RIGAKU/EFG XRT-100CCM X-ray diffractometric system (Freiberg Instruments, Freiberg, Germany) [20]. The X-ray diffraction topograms were obtained with the use of the microfocus X-ray source. The divergent X-ray beam of characteristic Cu_Kα_ radiation was applied using the PANalytical Microfocus DY0601 diffractometer (Malvern Panalythical, Almelo, The Netherlands). The topograms from the surface P were obtained for 113 and 002 type reflections. The methods of obtaining and interpretation of the topograms are described in Ref. [21,22,23,24,25]. 

## 3. Results and Discussion

Figure 2 shows the dendritic structure of part h visualized on section P (Figure 1b). The areas of different morphologies of the dendrite array are visible. In the first area marked as A (Figure 2a,b) and located in the upper left fragment of the image, the most common morphology of the dendrite arm arrangement appears, represented by four-petal flowers-like shapes. 

At some fragments of area A the dendrites are arranged in chains situated along the directions p and q of the secondary dendrites. The exemplary chains are marked in Figure 2a,b as a_1_–a_3_ and b_1_,b_2_. The subarea that represents the continuer extension (CE) with a similar dendrite array also belongs to the area A. However, in the centre of the CE, where the selector extension (SE) subarea is located, the chains are less often observed.

The specific dendrite chain is visible in the left vertex of the area A (c^CE^—Figure 2a,b). In this area the dendrite chain morphology is different than the morphology of chain types a and b. Therefore, such morphology should be specified separately rather as the single secondary dendrite c^CE^ parallel to the q direction (Figure 2b) from which the arms grow densely in the direction p, perpendicular to q. The distance between these arms is much smaller than that between the secondary dendrite arms parallel to the q direction, belonging to the chains a_1_–a_3_ type (Figure 2b). 

In area B, finer dendrites with smaller inter-dendritic distances occur compared to area A (Figure 2b,c). Additionally, in the upper right fragment of the P microsection (Figure 2c), the T area with a long dendrites c_1_–c_3_ similar to c^CE^ can be distinguished. These dendrites are almost parallel to the q direction. In the upper fragment of the T area, two long dendrites e_1_ and e_2_ almost parallel to the p direction are observed. The dendrite e_1_ tangentially contacts the side surface of CB3 and CB2 at the points U_3_ and U_2_ (Figure 2c), respectively, and at these points the direction changes. This dendrite belongs to both areas T and B. The dendrite e_2_ is curved and contacts the surface of CB3 at point W. Its curvature is not related with a continuous change of a direction between the points H and W, but with a step change in the dendrite direction—resulting in the creation of several straight sections (Figure 2c). The arm e_2_ is related to the c_1_–c_3_ dendrites arranged perpendicular to it. As a result, the T area consists of the fan-like arrangement of the c_1_–c_3_ dendrites. The morphology of the dendrite array in the T area indicates a lateral growth of the e_1_, e_2_ and c_1_–c_3_ dendrites, perpendicular to the *Z* axis of the root (Figure 1a). 

In area B the chains of the dendrites and single dendrites parallel to the p direction are visible. For both areas A and B, the chains and single dendrites in the direction p are more common than in the direction q. 

The presence of the e_1_, e_2_ and c_1_–c_3_ type of dendrites in the T area suggests their lateral growth at the level of the cross-section P, above the plane of the continuer with the root connection (Figure 1d). In area A, the phenomenon is rare and takes place, for example, for the arm c^CE^ only. As stated in Ref. [11,24], each of the dendrite chains of a_1_–a_3_ type, visible on the P surface, is the result of the growth of a set of tertiary dendrites in the Z direction from the laterally growing single secondary arm below the P surface which was named as the leading arm. In the SE subarea the dendrites grow directly from the selector (three double arrows, Figure 1c), that is why the chains occur less often. The areas B and T usually have a finer dendritic structure than area A (Figure 2c). It means that in areas B and T the local growth rate is higher. The long dendrite observed in the B and T areas grow laterally on the P surface. Such dendrites were not observed in area A. It means that in area A the dendritic structure was not created by the lateral growth. The analysis of the heterogeneity of the dendrite array of the upper surface of part h (surface P) shows that the crystallization is not steady over the entire surface P. The crystallization at the level of the P surface takes place by two mechanisms: the growth of the dendrites along the *Z* axis and lateral growth in perpendicular directions lying on the P surface. At the level of the P surface in area A, the dendrite array morphology is the same as in the SE or CE subareas in which the dendrites growth has steady character. This means that the dendrites grow in this subarea under steady-state conditions too. However, in the T area the dendrites grew laterally. This means that the growth has an unsteady character. In the B area both types of growth occur. Therefore, it should be assumed that in this area the growth is also unsteady. It follows that the crystallization front is not parallel to the P surface and is curved.

**Figure 3:**
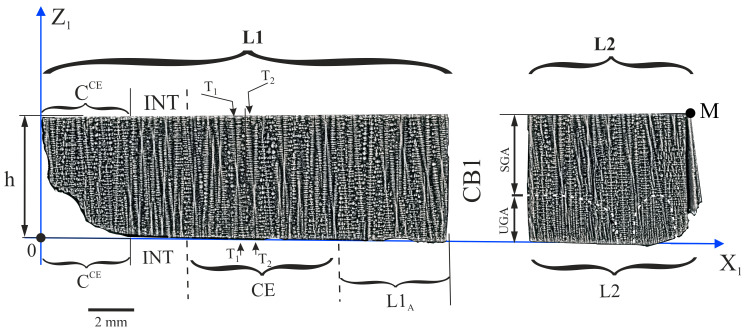
The dendritic structure of the h part visualised on the longitudinal section L (plane Z_1_X_1_). L1 and L2 are the fragments of the section separated by cooling bore 1 (CB1) (Figure 1d).

**Figure 4:**
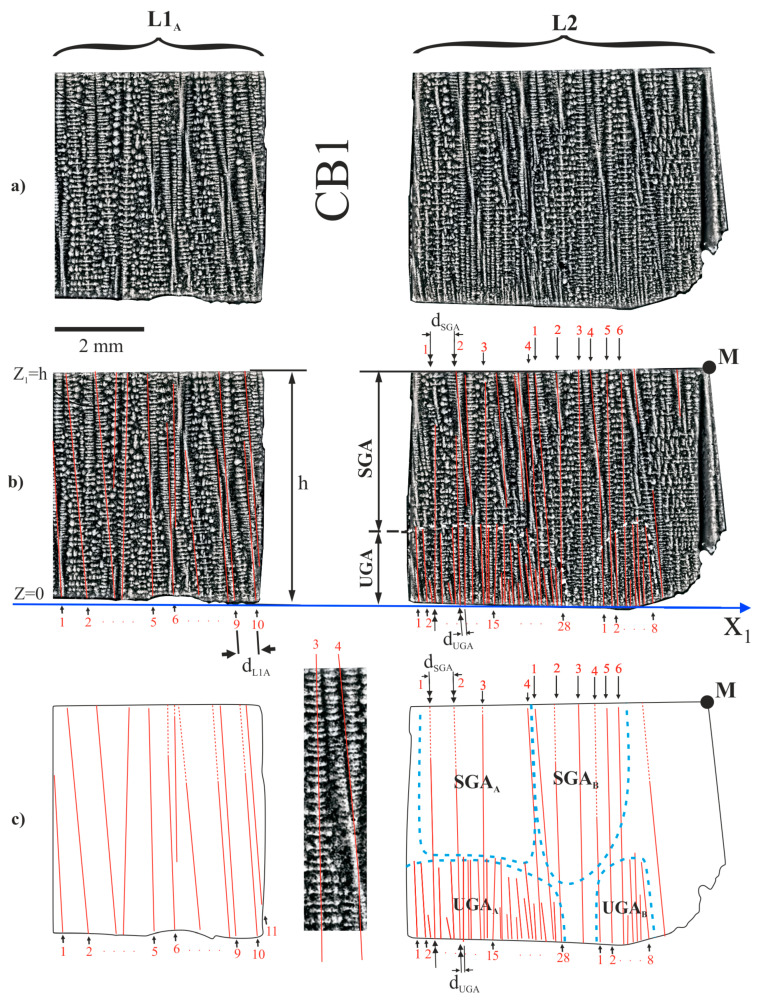
The dendritic structure of the h part of the root in the subarea L1_A_ and fragment L2 separated by CB1 (**a**,**b**) and scheme of the arrangement of the dendrites (**c**).

The dendrite array of the h part of the root was additionally analysed by examining the longitudinal section of the plane Z_1_X_1_ (Figure 1d, Figure 3 and Figure 4). The dendrite array was analyzed for the L1 and L2 fragments, separated by the cooling bore CB1 (Figure 3). The L2 fragment is also marked in Figure 2. The L1 fragment in Figure 3 includes the subareas indicated from left to right as: C^CE^, INT, CE, and L1_A_. Generally, in the L1 fragment the vertical dendrites are visible, but in subarea C^CE^ an additional horizontal short dendrite—type c^CE^—visible in Figure 2a,b, can be observed. Close to the C^CE^ subarea in Figure 3, the INT subarea with almost vertical dendrites—visualized as hourglasses—is visible. In the CE subarea, which is the continuer extension area, the dendrites visible as “hourglasses” “pass” through the entire thickness of the h part (e.g., T_1_ and T_2_ dendrites, Figure 3). This means, that in the CE subarea, the dendrites grow in the Z direction in a steady condition over the entire thickness of part h. This growth does not occur for the INT subarea, and the number of dendrites at the lower surface of part h is higher than at its upper surface. The inter-dendritic distance at the upper surface of the INT subarea is similar to that in the whole CE subarea. On the right side of the CE area the L1_A_ subarea can be distinguished, the structure of which is generally similar to the structure of the CE subarea. The location of the L1_A_ subarea in part h is also shown in Figure 1d. Furthermore, the subarea L1_A_ in Figure 3 is related to area A in Figure 2 and fragment L2 is generally related to area B (apart from the small L2 fragment located near the CB1 wall).

The structure of the dendrite array of the L1_A_ subarea near the upper (Z_1_ = h) and lower (Z_1_ = 0) surfaces is about the same (Figure 4). However, near the lower surface of the L2 fragment the structure is different than near its upper surface. The dendritic structure near the upper surface, in the subareas marked as SGA_A_ and SGA_B_ (Figure 4b,c), is similar to that in the CE subarea. This means, that in the SGA type subareas, the character of the dendrites’ crystallization is steady. In the two UGA_A_ and UGA_B_ subareas, marked in Figure 4b,c, the distance d_UGA_ between the neighbouring dendrites is smaller than the distance d_SGA_ in the SGA subareas. This means that UGA type subareas were crystallized in an unsteady condition at higher rates than the SGA type subareas. 

The dendritic structure near the lower surface (Z_1_ = 0) for the L1_A_ subarea does not differ significantly from the structure near the upper surface (Z_1_ = h). Since L1_A_ is the subarea of area A in Figure 2a, it can be concluded that the entire area A near Z_1_ = h crystallized at steady state conditions.

The dendritic structures of the L1_A_, SGA and UGA type subareas allows to define the interdendritic distance. The interdendritic distance, described as the linear arms spacing (LAS), has been defined earlier in Ref. [23]. The average value of the LAS was determined based on the scheme presenting the arrangement of the dendrite arms (Figure 4c). In Figure 4c, the almost vertical dendrite arms are presented as the numbered straight lines. In the case of the UGA, subareas’ dendrites are visualized as a straight lines starting at the bottom surface of the root, i.e., at the plane with the coordinate Z = 0. Therefore, determining the number of the dendrites and the LAS is quite simple and can be defined as the number of dendrites growing from the bottom surface. 

However, the selection of the dendrites in the SGA type subareas or in the L1_A_ is difficult because in these subareas the dendrites are visualized in the form of “conifer” with the small horizontal branches or “hourglasses” (dendrite 3 and 4 from the SGA_B_ in the insert of Figure 4c). In this case the criterion for selecting the dendrite lines was as follows: the lines pass, generally, through the axes of symmetry of the “hourglasses” or “conifers”. In the SGA type subareas the LAS, denoted in Figure 4b,c as d_SGA_, is evidently longer than in the UGA type subareas, e.g., d_SGA_ > d_UGA_ (Figure 4). This type of relation between the LAS also results from the analysis of the data presented in Table 1. The LAS of each area was calculated for dendrites numbered in Figure 4b,c. It can be also observed that the LASs in the L1_A_ subarea are comparable in terms of margin of error to the LAS of SGA_A_ and the SGA_B_ subareas of the L2 fragment (Table 1). Additionally, from Figure 4c it may be observed that only some dendrites (double arrows—Figure 4b,c) grow from the UGA to SGA subareas. This may be related to the effect of competitive growth of the dendrites. The upper fragments of the h part, denoted in Figure 4c as the SGA_A_ and SGA_B_ represent area A shown in Figure 2b, which is the area of steady dendrite growth. The lower UGA type subareas, described as the UGA_A_ and UGA_B_, represent areas of unsteady dendrite growth. 

**Figure 5:**
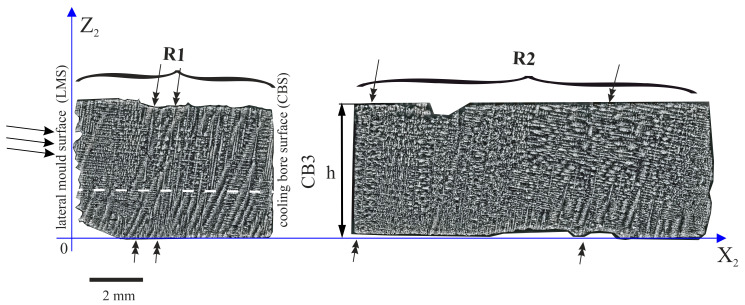
The dendritic structure of part h of the root, visualized on the longitudinal section R (plane Z_2_X_2_). R1 and R2 are the fragments of the section separated by the cooling bore 3 (CB3).

Figure 5 shows the dendritic structure of the h part, visualized on the surface of the Z_2_X_2_ plane (Figure 1d). It can be observed that a set of lateral dendrites grows from the lateral mould surface (LMS, Figure 2c) in the direction marked by arrows as the R1 fragment in Figure 5. Because the Z_2_X_2_ plane is parallel to the p direction (Figure 2c) those dendrites are visualized on the Figure 2 as the dendrites parallel to the p. 

**Figure 6:**
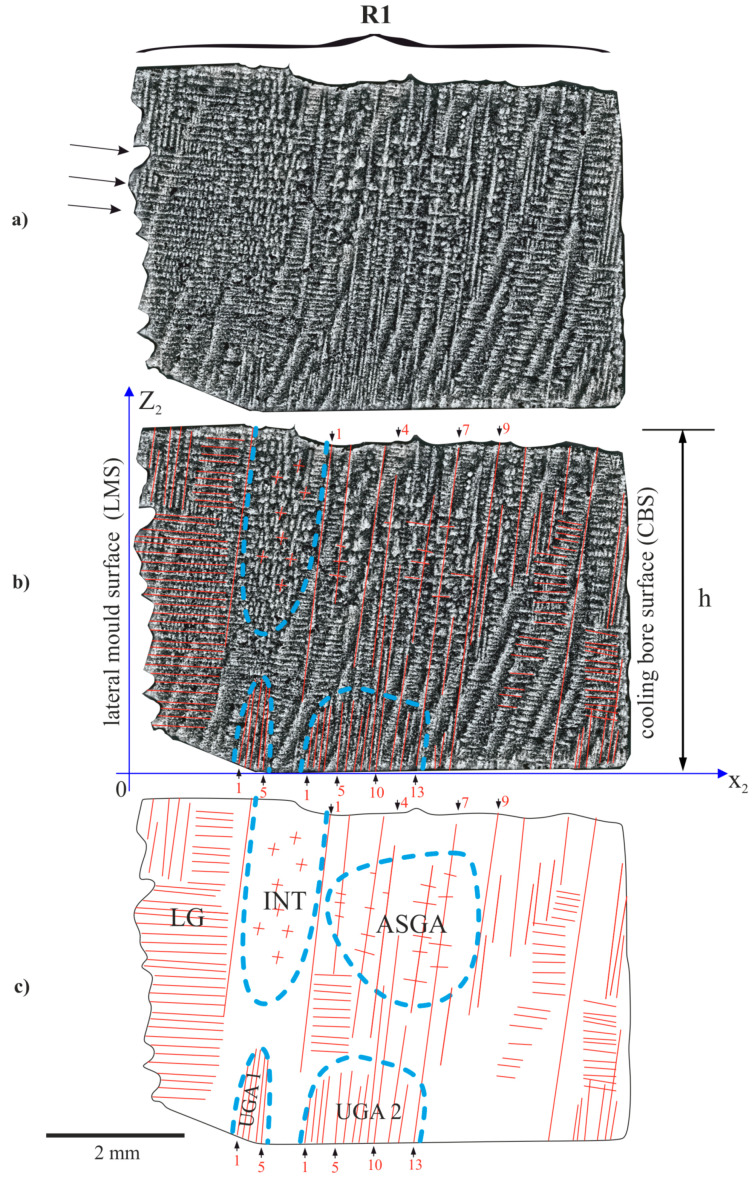
Dendritic structure of h part of the root in the R1 fragment (**a**,**b**) and scheme of dendrites arrangement (**c**).

The R1 fragment can be divided into five subareas (Figure 6): the lateral growth area LG, the unsteady growth subareas UGA1 and UGA2, the almost steady growth subarea ASGA and the interference subarea INT, where the images of vertically and horizontally growing dendrites overlap (Figure 6a–c). The shapes of the subareas of unsteady dendrite growth are similar to those which were found in the L2 fragment (Figure 4). The envelope of these areas has the shape of the curves with maxima (Figure 6c). However, in the ASGA subarea, images of horizontally growing dendrites overlap the images of the dendrites growing vertically. 

**Figure 7:**
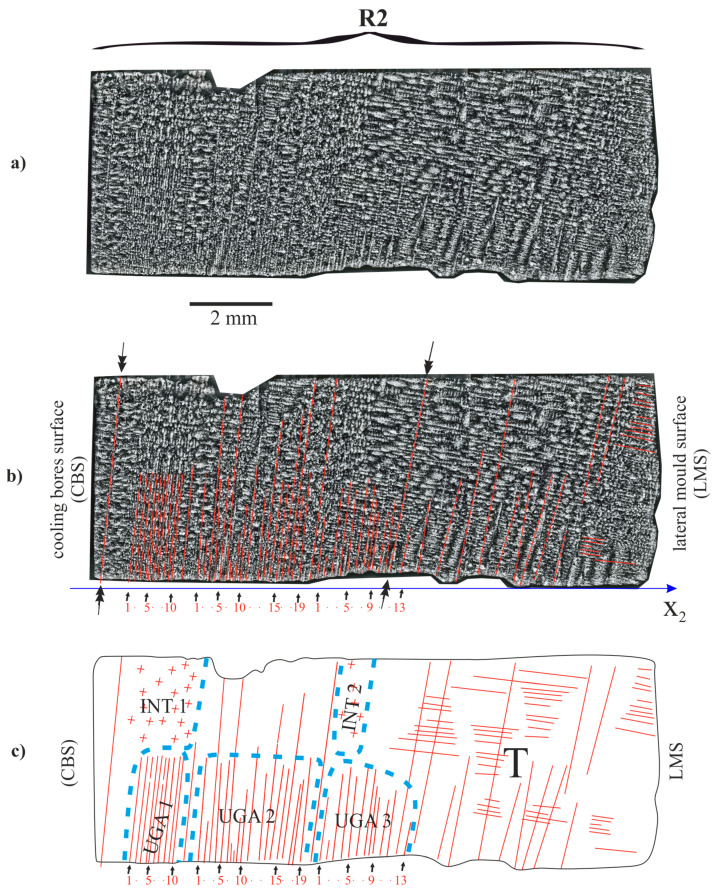
Dendritic structure of part h of the root in the R2 fragment (**a**,**b**) and scheme of the arrangement of the dendrites (**c**).

The LAS for the ASGA subarea is 0.32 mm (Table 2). This value was determined for nine vertically growing dendrites marked in Figure 6c. In the subareas UGA1 and UGA2, where the fine dendrites almost parallel to the *Z* axis occur, the LASs for these dendrites are low and reaches about 0.12–0.14 mm (Table 2). The LAS of the particular areas was calculated for the dendrites numbered in the Figure 6b,c and Figure 7b,c. In Figure 6, on the left side of the INT subarea, the dendrites are located almost horizontally and lie partially on the plane of the surface of the R1 fragment. In the INT subarea, as a result of the horizontal and vertical arm interference, the dendritic structure contains both short horizontal and vertical dendrite fragments. The dendrites of the LG subarea visible in the Figure 6 are also presented in Figure 2 as a dendrite parallel to the p direction.

The linear arm spacing of the dendrites in the UGA1, UGA2 and UGA3 subareas of the R1 and R2 fragments and the ASGA subarea of the R1 fragment are presented in Table 2. For all of the UGA type subareas of the R2 fragment, the LAS varied from 0.12 to 0.18 mm. For the ASGA subarea, the LAS value was significantly higher (0.32 mm, Table 2). In the subarea ASGA of the R1 fragment (Figure 6), specific competition between the almost vertically and almost horizontally growing dendrites occurs. A similar phenomenon also occurs in the area T of the R2 fragment (Figure 7c). However, for this area, the LAS of vertically growing dendrites (Table 2) was not determined because almost all dendrites grow horizontally. However, the single dendrites marked in Figure 7b by the double arrows, grow from the bottom surface of the mould to the top surface of part h.

Inside the subarea ASGA of the R1 fragment, specific competitive-like growth occurs. The almost horizontal arms growing from the lateral mould surface (LMS), the arms growing from the lateral cooling bore surfaces (CBS, Figure 6b,c) and the almost vertical dendrites growing from the almost horizontal leading secondary dendrites (not visible in Figure 6 and Figure 7)—localized near the bottom surface of the casting mould—are visible in the Figure 6. The subareas UGA1 and UGA2 consist of a group of very tightly arranged short dendrites. The shape of the subareas is complex and limited by the envelope with a maximum. Similar subareas are also found in the R2 fragment (Figure 7). In Ref. [11,24], it was stated that each group of the dendrites of UGA type subarea is created by the tertiary dendrites that grow almost parallel to the *Z* axis from a single secondary dendrite growing laterally near the bottom surface of the root. 

**Figure 8:**
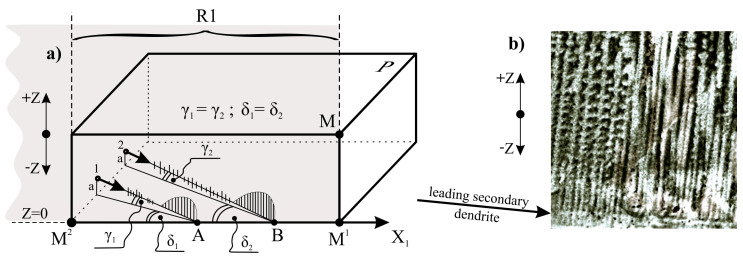
Scheme of creation in UGA type subareas sets of tertiary fine dendrites near the points A and B (**a**); the exemplary dendritic structure in which the set of tertiary dendrites growing from secondary dendrite are observed (**b**). The angles γ_1_ and γ_2_ are equal and small (a few degrees or less) and are enlarged for figure clarity. The angles δ_1_ and δ_2_ are equal and small (several degrees or less) because the plane MM^1^M^2^ of longitudinal section, is almost parallel to the secondary dendrites.

The reasons for the creation of the groups of fine tertiary dendrites may be explained as follows. Each group can grow from the single secondary dendrite growing almost parallel to the X_1_ axis (Figure 8). Such a secondary dendrite can be said to be leading. When the leading dendrite approaches the bottom surface of the root (plane Z = 0, Figure 8), the rapid growth of the densely arranged tertiary arms occurs. A similar phenomenon was described in Ref. [25]. According to this phenomenon, thin secondary dendrites grew in the group of small spacing near the bottom surface of the mould. Due to the fact that the bottom surface of the mould may be achieved by the several leading dendrites (for example, dendrite 1 and 2; Figure 8), several groups of fine tertiary dendrites are created near point A and B of the bottom surface. In addition, due to the fact that the surface of the longitudinal section R was similar to the directions of the secondary dendrites, the growth of the tertiary dendrites could be observed in the R section.

Since the lateral growth rate is an order higher than the withdrawal rate of the root out from the heating area of the Bridgman furnace [25], the growth takes place as if the removing the root from the heating area was “frozen”. It means, that the tertiary dendrites can grow temporarily not only in the +Z direction but additionally in the opposite (−Z) direction (Figure 8). However, since the value is small, the dendrites growing in the −Z direction will be very short. This can be seen that in some areas of the root near, the casting mould surface (Figure 8b). The growth of the tertiary dendrites stops at points A and B because the growth of the leading secondary dendrites stops. Near points A and B, the growth of the dendrites in the −Z direction stops, while in the +Z direction it intensifies. As a result, sets of longer tertiary dendrites are formed near points A and B, limited by the envelopes with local maxima. Such the sets of the dendrites are visible in the UGA1, UGA2 and UGA3 subareas in Figure 6 and Figure 7 or in the UGA_A_ or UGA_B_ in Figure 4.

The heterogeneity of the dendritic structure of the h part may be related to the local changes in the crystal orientation of the dendrites. Since the dendritic structure of the P surface is finer for the B and T areas compared to area A (Figure 2), it can be concluded, that the growth rate of the dendrites in the areas B and T is higher compared to area A. In areas B and T, the dendrites’ growth is affected by the lateral mould surfaces and surfaces of the ceramic cores of casting mould may occur. The accelerated process of dendrite growth occurs in the UGA type subareas (Figure 4, Figure 6 and Figure 7) which are areas of unsteady longitudinal almost-vertical growth. In the SGA type subareas (Figure 4) and in the almost whole upper surface of the L1 fragment (Figure 3) the process of dendrite growth almost vertical in the Z direction takes place in the steady-state regime. However, in area T, far from the selector extension area, the dendritic growth has an unsteady and almost lateral character.

In the L2 and R1 fragments (Figure 4 and Figure 6), local heat dissipation through the ceramic cores placed in the CB1–CB3 bores, play a fundamental role in the local increase in the dendrite growth rate during the initial stage of crystallization. The R2 is the fragment with a structure that suggest the fast-crystallizing, which may lead to the formation of the subgrains of high crystal misorientation. 

**Figure 9:**
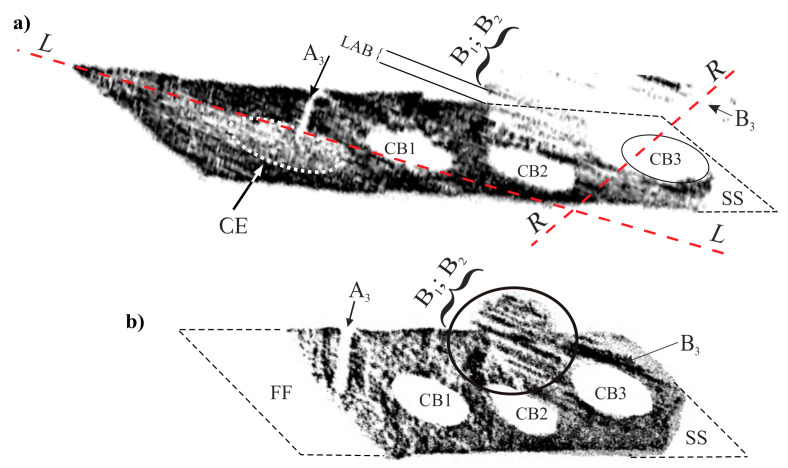
X-ray diffraction topograms obtained from the P surface using the 002 Cu_Kα_ reflex (**a**) and 113 Cu_Kα_ reflex (**b**). L and R—location of longitudinal sections.

To verify the above considerations, the X-ray diffraction topograms from the entire P surface were obtained. Figure 9 shows such topograms, obtained using the 002 and 113 Cu_Kα_ reflection. In the topograms, the areas of lacking contrast correspond to the cooling bores CB1—CB3 and areas of increased and decreased contrast in the shape of bands are visible. The B_1_–B_3_ contrast bands that are shifted relative to the rest parts of topograms, may be observed for area T of the P surface. It means that in these areas of the high misoriented subgrain of, a band-shape exists. The direction of these bands is consistent with the direction of the q dendrites visible in Figure 2. 

The shape of the outline of the topograms differs from the shape of the outline of the P plane from which it was obtained. The differences result from the diffraction geometry, i.e., from the slope of the diffraction beam and the (002) and (113) diffraction planes, relative to the studied surface. The shifted bands of B_1_–B_3_ areas are present near the cooling bores CB2 and CB3. The bands of lacking contrast between the shifted fragments of the topograms represent the low-angle boundary (LAB). 

In addition to the areas of lacking contrast related to the local shifts in the topograms, the regions A_3_, SS and FF are visible. These regions are formed when the Bragg condition is not satisfied [20] and there is no X-ray reflection. The location of the A_3_ area on the topogram corresponds to the location of the a_3_ dendrite chains on the P surface (Figure 2a,b). This means that all dendrites of the chains are highly disoriented with the rest of the dendrites. The leading secondary dendrite, from which the chain of the dendrites grew, was probably accidentally rotated by a fairly large angle. The location of the SS area (lack of contrast) corresponds to the right lower fragment of area T on the P surface (Figure 2a,c). The image of the dendritic structure in this area shows the c_1_–c_3_ dendrites growing laterally from the lateral mould surface. The crystal misorientation of this area is so large that the Bragg condition is not satisfied, and X-ray beam reflection does not occur. The contrast bands B_1_ and B_2_ in the topogram correspond to the chains of the dendrites similar to b_1_ (Figure 2c). Band B_3_ corresponds to the side branches of the dendrites parallel to b_1_, growing from the dendrite e_1_ or e_2_. The lack of contrast for the area FF is a result of the absence of proper Bragg conditions for reflection 113 corresponding to this area. Details on how the X-ray diffraction topograms were obtained and ineterpreted are described in Ref. [26].

The above-mentioned shifts of B_1_–B_3_ areas in the topogram indicating the crystal misorientation, most often occur near the cooling bores or near the surface of the mould. The local heat transfer is faster towards the mould wall and could be the reason for the rapid growth of the dendrites parallel to the heat dissipation direction. The high rate of growth is the cause of the crystal misorientation. The CE subarea on the topogram from Figure 9a has a uniform contrast that suggests no misorientation defects.

The growth of the dendrite is often limited and changed by the mould walls, resulting in the bending of the dendrites [10,12,27,28,29]. The bending is observed in the areas localized near the mould surface or in the areas where the step change geometry of cast occurs, for example in the root near the continuer-root connection. 

The selector location on the transverse section of the root has a significant influence on the dendrites growth and the dendritic array of the blades. In the studied root of the turbine blade, the selector was located asymmetrically relative to the centre of the root transverse-section and shifted towards the edge of the blade (Figure 1c). The continuer extension (CE) subarea of the root is free of the essential defects of the misorientation character or heterogeneity of the dendrite array that appear in the other areas of the root. The highest concentration of defects, especially subgrains, appears a long distance away from the continuer extension (CE) subarea. There are a few reasons for the formation of the dendrite array heterogeneity at the initial stage of the crystallization in the root (i.e., in part P) of the cored SX-blades.

**Figure 10:**
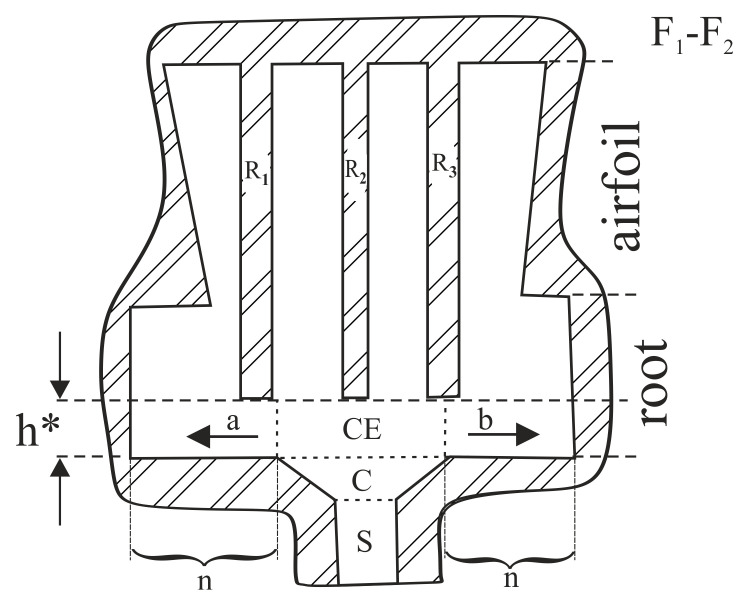
Section F1–F2 (Figure 1b) of the casting mould with R_1_, R_2_ and R_3_ ceramic cores, visualization of the shape of the mould that enables undisturbed lateral growth of dendrites in a and b directions inside fragment h* of the root. S and C—selector and continuer of selector. CE—continuer extension area of root.

The first reason is the strongly asymmetrical location of the selector and continuer (Figure 1b). The second is related to the location of the mould on the chill-plate and the mould dimensions in comparison to the diameter of the chill-plate. Due to the relatively small diameter of the chill-plate (D_CP_ = 20 cm) in relation to the *l* size (*l* = 60 mm) of the root, the casting mould was located on the chill-plate in a such way that the longer edge of the root (edge with length *l*) is not aligned with the radius R but with the tangent to the circumference of the chill-plate (Figure 1c). This probably caused significant heterogeneity of the temperature distribution in the cast. The additional negative aspect is that the ceramic cores of the cooling bores pass through the whole mould and connect to the bottom surface of the casting mould. As a result, the cores were an obstacle to the lateral growth of the dendrites in the initial stage of crystallization, during the transition of the crystallization front from the continuer to the root. To prevent the formation of the dendrite array heterogeneity, the single-crystalline cast of the blades must include the root fragment h*, which should not contain the cooling bores (Figure 10). This fragment would allow for unsteady and fast undisturbed lateral growth of the dendrites from the continuer extension (CE) area to the other areas of the root. This fragment should be cut off in the next stages of blade production. As a result, fewer defects will be generated in the h* fragment (Figure 10) and inherited by the areas of the cast that crystallize later (the root and the airfoil). Additionally, the continuer should be placed in the middle of the cross-section of the root. This will reduce the distance *n* (Figure 10) in which rapid lateral growth occurs, and will reduce the number of defects in the h* fragment. The thickness of h* must be greater than or equal to the fragment of unsteady growth with the thickness h which was about 5 mm for the tested casts.

## 4. Conclusions

The dendrite array of the root part of the cored turbine blades—created during the initial stage of crystallization—is not homogenous. The heterogeneity of the dendrite array and the crystal misorientation of the local areas results in the formation of subgrains and low-angle boundaries, which are mostly created near the cooling bores and at a considerable distance from the selector continuer extension area of the root. 

In the side of the continuer extension subarea—and its close proximity—and closer to the axis of the growth chamber (area A in Figure 2), there are single dendrites visualized in the form of four-petal flowers and chains of dendrites. The single dendrites and the chains of the dendrites grew in a direction almost parallel to the *Z* axis of the root. In area A, the dendrites array is created without the lateral growth of dendrites and crystallization has a steady character. In the area located near the wall of the growth chamber (B area, Figure 2) the dendrites grow at a higher rate than in the area of steady growth (A area). This means that, in this area, the growth of the dendrites has an unsteady character. In the area localized far from the continuer extension (T area, Figure 2) the dendrites grow laterally and the crystallization has an unsteady character. It follows, that during the initial stage of crystallization near the continuer–root plane connection, the crystallization front is not parallel to the horizontal transverse section of the root and is bent in a complex manner.

The heterogeneity of the dendrite array, resulting from the heterogeneity of the dendrites growth during the initial stage of crystallization, occurs in the root most frequently at the greatest distance from the selector extension. Both the external and internal mould wall have a high influence on the array heterogeneity. 

On the basis of the above-described study, the following conclusions can be drawn: in the single-crystalline cored turbine blade casts, the selector continuer or selector should be connected with the root through a fragment which should not contain the cooling bores. In this fragment, dendrites will grow laterally, unsteadily and quickly—without obstacles in the form of the surfaces of the cores of the casting mould—from the selector extension area to the other areas of the root. This fragment should be cut off and rejected in the next stages of the blade production. Additionally, the selector should be located symmetrically in the centre of the transverse section of the root (Figure 10). 

## Figures and Tables

**Table 1:** The values of the linear arms spacing (LAS) for both L1_A_ subarea of L1 fragment and subareas SGA_A_, SGA_B_, UGA_A_, UGA_B_ of L2 fragment.

L1_A_ Subarea	L2 Fragment	
0.43 ± 0.20 mm (calculated for 11 dendrites)	SGA_A_	0.39 ± 0.06 mm (calculated for 4 dendrites)
	SGA_B_	0.37 ± 0.12 mm (calculated for 6 dendrites)
	UGA_A_	0.12 ± 0.04 mm (calculated for 28 dendrites)
	UGA_B_	0.18 ± 0.09 mm (calculated for 8 dendrites)

**Table 2:** The values of linear arms spacing (LAS) for the subareas located inside both R1 and R2 fragments.

		R1 Fragment	R2 Fragment
ASGA		0.32 ± 0.08 mm (calculated for 9 dendrites)	not determined
	T		
UGA1		0.12 ± 0.04 mm (calculated for 5 dendrites)	0.12 ± 0.02 mm (calculated for 10 dendrites)
UGA2		0.14 ± 0.05 mm (calculated for 13 dendrites)	0.17 ± 0.06 mm (calculated for 19 dendrites)
UGA3		-	0.18 ± 0.05 mm (calculated for 13 dendrites)

## Data Availability

Data sharing is not applicable to this article.
